# Glucose Counteracts Isoprenaline Effects on Ion Channel Functions in Human-Induced Pluripotent Stem Cell-Derived Cardiomyocytes

**DOI:** 10.3390/jcdd9030076

**Published:** 2022-03-04

**Authors:** Lin Qiao, Xuehui Fan, Zhen Yang, Ibrahim El-Battrawy, Xiaobo Zhou, Ibrahim Akin

**Affiliations:** 1 Department of Cardiology, Angiology, Haemostaseology and Medical Intensive Care, Medical Faculty Mannheim, University Medical Centre Mannheim (UMM), University of Heidelberg, Theodor-Kutzer-Ufer 1-3, 68167 Mannheim, Germany; qiaolin3952@163.com (L.Q.); xuehui.fan@medma.uni-heidelberg.de (X.F.); zhen.yang@medma.uni-heidelberg.de (Z.Y.); ibrahim.elbattrawy2006@gmail.com (I.E.-B.); ibrahim.akin@umm.de (I.A.); 2 Key Laboratory of Medical Electrophysiology of Ministry of Education and Medical Electrophysiological Key Laboratory of Sichuan Province, Collaborative Innovation Center for Prevention of Cardiovascular Diseases, Institute of Cardiovascular Research, Southwest Medical University, Luzhou 646000, China; 3 European Center for AngioScience (ECAS) and German Center for Cardiovascular Research (DZHK) Partner Site Heidelberg/Mannheim, 68167 Mannheim, Germany

**Keywords:** diabetes, high glucose, Takotsubo syndrome, ion channel function, arrhythmia

## Abstract

Recent studies indicate that the disorder of glucose metabolism in myocardial tissue is involved in the development of Takotsubo syndrome (TTS). This study investigated the effects of a high level of glucose on the pathogenesis of TTS, focusing on the electrophysiological mechanisms. Human-induced pluripotent stem cell-derived cardiomyocytes (hiPSC-CMs) were treated with toxic concentration of isoprenaline (Iso, 1 mM) and a high level of glucose (22 mM) to mimic the setting of TTS and diabetes mellitus (DM). Iso prolonged action potential duration (APD) through enhancing the late sodium channel current and suppressing the transient outward potassium current (I_to_). However, a high level of glucose prevented the APD prolongation and the change in I_to_. High-level glucose reduced the expression levels of PI3K/Akt, β1-adrenoceptors, Gs-protein, and PKA, suggesting their involvement in the protective effects of high-level glucose against toxic effects of catecholamine. High glucose level did not influence Iso-induced ROS-generation, suggesting that the protective effects of high-level glucose against Iso did not result from changes in ROS generation. High-level glucose may protect cardiomyocytes from the toxic effects of catecholamine excess through suppressing β1-adrenoceptor-Gs-PKA signaling. DM may reduce the risk for occurrence of arrhythmias due to QT prolongation in TTS patients.

## 1. Introduction

Takotsubo syndrome (TTS), which is also known as “stress induced cardiomyopathy”, was first described in Japan in 1990. It is characterized by transient systolic dysfunction of the apical and middle segments of the left ventricle, with clinical features of myocardial infarction but without obstructive coronary artery disease (CAD) [1]. For a long time, the prognosis of patients with TTS was considered to be favorable. However, about 20% of TTS patients have severe cardiac complications in the acute phase, which is comparable with acute coronary syndrome (ACS) [2]. The critical complications associated with TTS include atrial fibrillation, ventricular arrhythmias, thrombus formation, and heart failure (HF) [3,4,5,6,7,8,9]. Acquired long QT syndrome (LQTs) was described in almost 50% of TTS patients and can lead to life-threatening arrhythmias (ACS) [2]. Although the exact mechanism of TTS is still unknown, it is generally accepted that the development of the disease is associated with catecholamine excess.

Several researchers reported that TTS patients have a lower prevalence of diabetes mellitus (DM). A review study found that the prevalence of DM in patients with TTS to be around 10.2–17%, which was lower than the 26.9% in the general population reported in the National Health and Nutritional Examination Survey (NHANES) [10]. Another recent register study confirmed the finding: compared with ACS patients (19.8%), the incidence of DM in TTS (6.5%) was significantly lower [11]. Diabetes can cause autonomic neuropathy, and it may have a protective effect on TTS under conditions of emotional or physical stress. However, whether high glucose can exert protective effects against catecholamine excess is still unknown.

For exploring the pathogenesis of TTS, good experimental models are required. Animals and animal cells, human cell lines such as HEK293, and HeLa cells have been used in some research. Due to the difference between animals and humans or noncardiac and cardiac cells, studies in human cardiomyocytes have advantages. Human-induced pluripotent stem cell-derived cardiomyocytes (iPSC-CMs) for the study of human heart diseases possess advantages such as: (1) hiPSC-CMs are from human cells, excluding possible differential effects resulting from differences between humans and animals; (2) hiPSC-CMs are cardiomyocytes similar to human native cardiomyocytes, avoiding the limitations of non-cardiac cells, which lack some molecules and signaling that are important for heart functions; (3) hiPSC-CMs have been shown to be able to recapitulate some cardiac disorders, such as long QT syndrome, short LQ syndrome, Brugada syndrome, arrhythmogenic right ventricular cardiomyopathy, and Takotsubo syndrome [12,13,14,15,16]; (4) hiPSC-CMs possess electrophysiological and pharmacological features similar to human native cardiomyocytes; (5) studies using hiPSC-CMs can provide useful preclinical data for further clinical studies without animal sacrifice and can overcome the availability limitation of cardiomyocytes directly from the heart of human patients.

Recently, our group successfully used hiPSC-CMs to investigate the toxic effects of a high concentration of isoprenaline (Iso) that mimics the setting of TTS [16]. Other studies showed that a DM-model of hiPSC-CMs accurately produced features of DCM in vitro, including cellular hypertrophy, lipid accumulation, and evidence of oxidative stress [17]. Therefore, we used hiPSC-CMs to investigate the effects of a high level of glucose on the pathogenesis of TTS.

## 2. Methods

### 2.1. Ethics Statements

The skin biopsies from three healthy volunteers who gave a written declaration of consent were used in our study. The research was approved by the Ethical Committee of the Medical Faculty Mannheim, University of Heidelberg (approval number: 2018-565N-MA), and was conducted according to the specifications of Helsinki Declaration of 1975, with the revised version of 1983.

### 2.2. Generation of hiPSCs

The generation of hiPSCs was performed by Dr. Cyganek’s group (the Stem Cell Unit, Clinic for Cardiology and Pneumology, University Medical Center Göttingen, Germany). A detailed description of the generation of the three hiPS cell lines is provided in our recent publications [13,18].

### 2.3. Generation of hiPSC-CMs

The frozen aliquots of hiPSC lines from the three healthy donors were thawed and cultured without feeder cells and differentiated into cardiomyocytes (hiPSC-CMs) as described previously [13,14,16]. The hiPSC-CMs were then cultured in maintenance media at least to day 40–60. After the cells were 40 days old and contractible, they were used for various experiments.

### 2.4. Polymerase Chain Reaction Assays

Quantitative polymerase chain reaction assays (qPCR) were performed following the protocol. Using a RNeasy mini kit (Qiagen, Hilden, Germany), the total RNA was first extracted. The RNA was then transcribed into cDNA on a Stratagene MX 3005P Real-Time Cycler (Stratagene, La Jolla, CA, USA) with the use of High-Capacity cDNA Reverse Transcription Kit (Applied Biosystems, Forster City, CA, USA). After that, real-time quantitative PCR analysis was performed on StepOnePlus Real-Time PCR Systems (Thermo Fisher) with the use of SYBR Green PCR kit (SibirRox Hot Mastermix, Bioron, Germany). The expression of mRNA of target genes relative to housekeeping gene GAPDH in samples of treated and untreated (control) cells was calculated with ΔΔCT method, which was based on the cycle threshold (CT), where the relative expression factor = 2^−Δ(ΔCT)^, ΔCT = CT gene of interest—CT GAPDH, and Δ (ΔCT) = ΔCT treated—ΔCT control. The requirement for the ΔΔCT in the calculation method is a doubling of the DNA in each PCR cycle [19]. The experiment with control and treated groups was repeated at least three times independently, and cDNA was measured from three cell culture wells in each experiment to ensure reproducibility.

### 2.5. Immunofluorescence Staining

Immunofluorescence staining was performed for detecting proteins. Images were collected using the fluorescence microscope Leica DMRE DFC3000G. The primary antibodies were monoclonal anti-α-actinin antibody (A7811, 1:200; Sigma-Aldrich, Merck KgaA, Darmstadt, Germany), anti-cardiac troponin T antibody (ab8295, 1:200; Abcam, Cambridge, UK), anti-Kv4.3 (KCND3) antibody (APC-017, 1:400; Alomone Labs, Jerusalem, Israel), and anti-beta 1 adrenergic receptor antibody (ab3442, 1:400; Abcam, Cambridge, UK).

### 2.6. ROS Production Measurement

2′,7′-Dichlorodihydrofluorescein diacetate (DCFH-DA, Sigma) was used to detect the production of intracellular reactive oxygen species (ROS). Briefly, cells were seeded in 35 mm dishes and cultured overnight. After drug treatment, cells were washed twice with PBS and loaded with 5 μM DCFH-DA solution in serum-free medium at 37 °C for 30 min in the dark. Cells were then washed with PBS solution and subjected to fluorescence measurement using a fluorescent microscope (BX51; Olympus Corp).

### 2.7. Patch-Clamp

This study employed the whole-cell recording technique to measure action potential (AP) and ion channel currents at room temperature, as described before [14,16]. Briefly, AP was measured in current-clamp mode with stimulating pulses (1 nA, 3 ms) at 1 Hz. Peak and late sodium channel currents were measured with pulses of 400 ms from −120 mV to 50 mV (in 5 mV steps) with a holding potential of −100 mV. Mean values of the currents at −40 mV were used for statistical analyses. The late I_Na_ at −40 mV was measured as the area under the current curve to zero line from 300 ms to 350 ms. TTX (30 µM) was applied to inhibit late I_Na_, and the TTX-sensitive current was analyzed as late I_Na_. 4-AP (3 mM) was applied to inhibit the transient outward potassium current (I_to_), and the 4-AP-sensitive current was analyzed as I_to_. NiCl_2_ (5 mM) was applied to inhibit Na/Ca exchanger current (I_NCX_), and the NiCl_2_-sensitive current was analyzed as I_NCX_. E-4031 (1 µM) was applied to inhibit rapidly activating delayed rectifier potassium channel current (I_Kr_), and the E-4031-sensitive current was analyzed as I_Kr_. Chromanol 293B (10 µM) was applied to block slowly activating delayed rectifier potassium channel current (I_Ks_), and the chromanol 293B-sensitive current was analyzed as I_Ks_.

### 2.8. Statistics

Data are shown as mean ± SEM (mean value and standard error of the mean) and were analyzed by SigmaPlot 11.0 (Systat GmbH, Düsseldorf, Germany). The Student’s *t*-test was used to compare two independent groups with normal distribution. One-way ANOVA with Holm–Sidak post-test for multiple comparisons was applied for parametric data in more than two groups. A value of *p* < 0.05 was assumed to be statistically significant.

## 3. Results

### 3.1. High Concentration of Glucose Counteracted Isoprenaline Effects on Action Potentials

**Figure 1:**
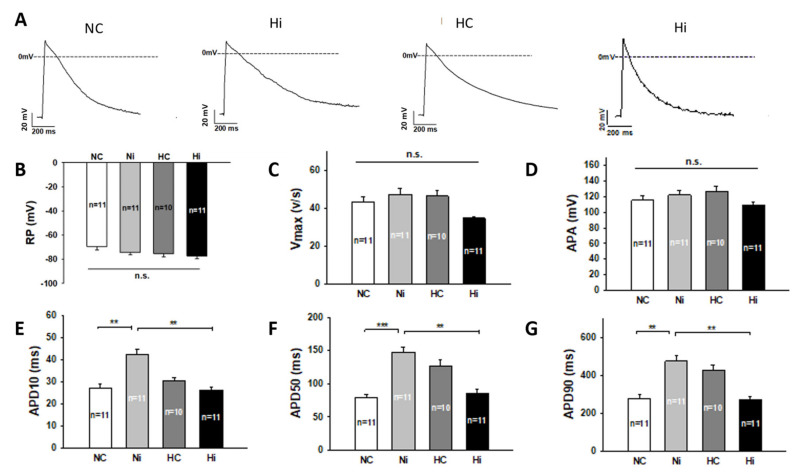
High level of glucose counteracted the effects of high-concentration isoprenaline on action potentials. “NC” represents data from hiPSC-CMs treated with normal level of glucose (5.5 mM). “Ni” represents data from hiPSC-CMs treated with normal level of glucose and high concentration (1 mM for 1 h) of Iso. “HC” represents data from hiPSC-CMs treated with high level (22 mM for 14 days) of glucose. “Hi” represents data from hiPSC-CMs treated with a high level of glucose and a high concentration of Iso. Action potentials (AP) were recorded at 1 Hz stimulation. (**A**) Representative traces of APs in different groups. (**B**) Mean values of resting potentials (RP). (**C**) Mean values of maximal depolarization velocity of AP (Vmax). (**D**) Mean values of action potential amplitude (APA). (**E**) Mean values of action potential duration at 10% repolarization (APD10). (**F**) Mean values of action potential duration at 50% repolarization (APD50). (**G**) Mean values of action potential duration at 90% repolarization (APD90). Data are presented as mean ± SEM and analyzed by one-way ANOVA with Holm-Sidak post-test. The cell number of every experimental group is given as “n”. ** *p* < 0.01, *** *p* < 0.001. “n.s.” means no significance.

To examine the influences of high level of blood sugar in diabetes on phenotypic feature of TTS, the hiPSC-CMs were pre-treated with a high level of glucose (22 mM) for 14 days to mimic the setting of diabetes and then treated with a high concentration of Iso (1 mM) for 1 h to mimic the setting of TTS with catecholamine excess. Treatment with normal level of glucose (5.5 mM) was used as control. Action potential (AP) properties of hiPSC-CMs in all groups were assessed (Figure 1). After the treatment, neither high-concentration Iso nor a high level of glucose changed the resting potential (RP), maximal depolarization velocity of action potential (Vmax), or amplitude of action potential (APA) (Figure 1B–D). High-concentration Iso prolonged the action potential duration at 90% repolarization (APD 90), the action potential duration at 50% repolarization (APD 50), and the action potential duration at 10% repolarization (APD 10) (Figure 1A,E–G), respectively. Likewise, a high level of glucose could also prolong the action potential duration (APD). However, a high level of glucose and high-concentration Iso together failed to show any effect (Figure 1A,E–G), indicating that a high level of glucose can counteract the effects of high-concentration Iso on Aps. Of note, the effects of a high level of glucose on APDs also disappeared, implying that high-concentration Iso can also counteract the effects of high-level glucose.

### 3.2. Effects of High Concentration Isoprenaline and High Level of Glucose on Ion Channel Currents in hiPSC-CMs

**Figure 2:**
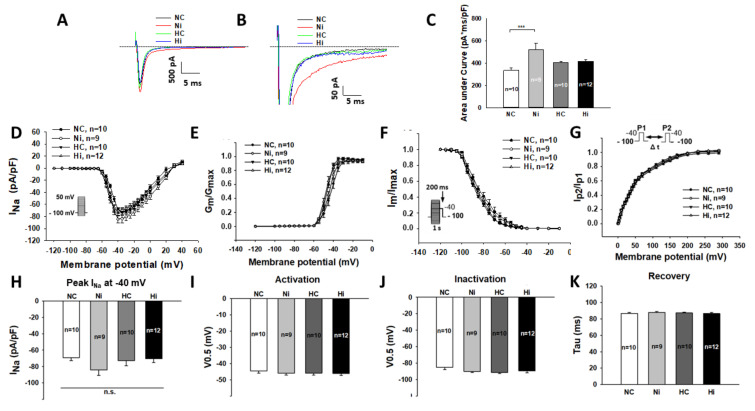
Effects of high-concentration isoprenaline and a high level of glucose on sodium channel currents. “NC” represents data from hiPSC-CMs treated with normal level of glucose. “Ni” represents data from hiPSC-CMs treated with normal level of glucose and a high concentration of Iso. “HC” represents data from hiPSC-CMs treated with a high level of glucose. “Hi” represents data from hiPSC-CMs treated with a high level of glucose and a high concentration of Iso. Sodium channel currents (I_Na_) were recorded using protocols shown in (**D**,**F**,**G**) (inset). The late I_Na_ was measured as the area under the current curve to zero line from 300 ms to 350 ms. (**A**) Representative traces of peak I_Na_ at −40 mV in cells from each group. (**B**) Representative traces showing late I_Na_ at −40 mV in cells from each group. (**C**) Mean values of late I_Na_ at −40 mV. (**D**) I–V curves of peak I_Na_ in cells of each group. (**E**) Activation curves of peak I_Na_ in cells of each group. (**F**) Inactivation curves of peak I_Na_ in cells of each group. (**G**) Peak I_Na_ curves of recovery from inactivation in cells of each group. (**H**) Mean values of peak I_Na_ at −40 mV. (**I**) Mean values of voltage at 50% activation. (**J**) Mean values of voltage at 50% inactivation. (**K**) Mean values of time constant (Tau) of recovery from inactivation. Data are presented as mean ± SEM and analyzed by one-way ANOVA with Holm–Sidak post-test. The cell number of every experimental group is given as “n”. *** *p* < 0.001. “n.s.” means no significance.

**Figure 3:**
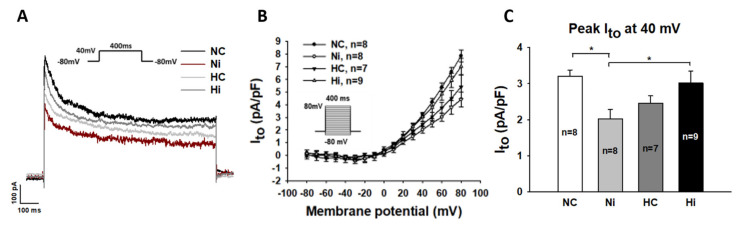
Effects of high-concentration isoprenaline and a high level of glucose on transient outward potassium current (I_to_). “NC” represents data from hiPSC-CMs treated with normal level of glucose. “Ni” represents data from hiPSC-CMs treated with normal level of glucose and a high concentration of Iso. “HC” represents data from hiPSC-CMs treated with a high level of glucose. “Hi” represents data from hiPSC-CMs treated with a high level of glucose and a high concentration of Iso. Transient outward potassium current (I_to_) was recorded using protocols shown in (**A**,**B**) (inset). (**A**) Representative traces of peak I_to_ at 40 mV in cells from each group. (**B**) I–V curves of peak I_to_ in cells of each group. (**C**) Mean values of peak I_to_ at 40 mV. Data are presented as mean ± SEM and analyzed by one-way ANOVA with Holm-Sidak post-test. The cell number of every experimental group is given as “n”. * *p* < 0.05.

The action potential duration was determined by different ion channel currents. To examine how high-concentration Iso and a high level of glucose influenced the AP duration in hiPSC-CMs, individual ion channel currents including I_Na_, I_Ca-L_, I_NCX_, I_to_, I_Kr_, and I_Ks_ were checked. No significant changes in the peak I_Na_ were detected in the four experimental groups (Figure 2A,D,H), while the late sodium current increased significantly in Ni (high level Iso) group when compared with NC (control) group (Figure 2B,C). Interestingly, a high level of glucose alone did not significantly change late I_Na_, but it prevented the effect of Iso on late I_Na_ (Figure 2B,C). A high level of glucose did not significantly reduce L-type calcium channel current (I_Ca-L_) (Figure S1). The transient outward potassium channel current (I_to_) was largely suppressed by high-concentration Iso and was less inhibited by a high level of glucose (Figure 3A–C). When cells were treated by high-level glucose plus Iso, the inhibitory effect was abolished (Figure 3). The enhancement of late I_Na_ and decrement of I_to_ were inconsistent with the prolongation of APD. The Na/Ca exchanger current (I_NCX_) was only slightly enhanced after treatments with high-concentration Iso or a high level of glucose, but the changes were not significant (Figure S2). The rapidly activating delayed rectifier potassium channel current (I_Kr_) and slowly activating delayed rectifier potassium channel current (I_Ks_) were not changed or significantly altered by either high-concentration Iso or a high level of glucose (Figure S3).

To explore mechanisms underlying the changes in ion channel current induced by high-concentration Iso and a high level of glucose, we checked the expression of the respective ion channels in hiPSC-CMs. The examined channel genes included SCN5A (coding Na^+^ channel, Nav1.5), SCN10A (coding Na^+^ channel, Nav1.8), CACNA1C (coding L-type Ca^2+^ channel), SLC8A1 (coding Na^+^/Ca^2+^-exchanger, NCX1), KCND3 (coding I_to_ channel, Kv4.3), KCNQ1 (coding I_Ks_ channel, Kv7.1), and KCNH2 (coding I_Kr_ channel, Kv11.1). The expression levels of SCN10A and SLC8A1 genes were elevated by high-concentration Iso but were reduced slightly by a high level of glucose (Figure S4B,D). On the other hand, the expression level of KCND3 was reduced by both high-concentration Iso and a high level of glucose (Figure S4E). Neither high-concentration Iso nor a high level of glucose significantly influenced the expression levels of SCN5A, CACNA1C, KCNQ1, or KCNH2 (Figure S4A,C,F,G).

On protein level, KCND3 protein was analyzed by immunostaining. The results show that KCND3 proteins in cell membranes were reduced in the presence of Iso and high glucose (Figure S5), which was consistent with PCR and patch clamp measurements.

### 3.3. High Level of Glucose Did Not Reduce the ROS Production Mediated by Isoprenaline

**Figure 4:**
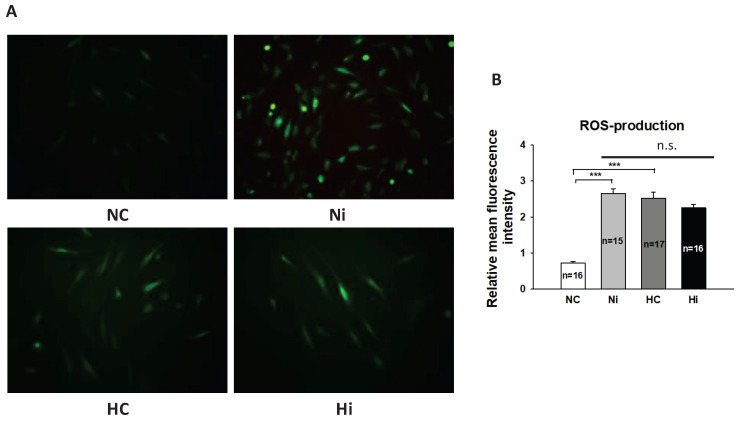
High-concentration isoprenaline and a high level of glucose induced ROS generation in hiPSC-CMs. Cells were incubated with 5 μM DCFH-DA solution in serum-free medium at 37 °C for 30 min in the dark in each cell group. A fluorescence microscope was used to evaluate the fluorescence intensity of cells. “NC” represents data from hiPSC-CMs treated with normal level of glucose. “Ni” represents data from hiPSC-CMs treated with normal level of glucose and a high concentration of Iso. “HC” represents data from hiPSC-CMs treated with a high level of glucose. “Hi” represents data from hiPSC-CMs treated with a high level of glucose and a high concentration of Iso. (**A**) Representative pictures showing the fluorescence intensity of cells in different groups. (**B**) Statistical analyses of fluorescence intensity representing levels of ROS. Magnification, ×400. Data are presented as mean ± SEM and analyzed by one-way ANOVA with Holm–Sidak post-test. The cell number of every experimental group is given as “n”. *** *p* < 0.001. “n.s.” means no significance.

It is known that high-concentration Iso and a high level of glucose can enhance the generation of reactive oxygen species (ROS) in cardiomyocytes [16,20], and ROS contributed to effects of Iso on channel currents [16]. Therefore, we assessed the ROS generation in hiPSC-CMs treated with or without high-concentration Iso and a high level of glucose. After the aforementioned treatment, fluorescence imaging of 2′,7′-dichlorodihydrofluorescein diacetate (DCFH-DA) was used to assess ROS level. It was observed that fluorescence intensity was very weak in cells without treatment, but it was significantly intensified by Iso and high glucose treatment (Figure 4) in hiPSC-CMs, which indicated elevation of ROS generation. Strikingly, a high level of glucose did not significantly reduce Iso-induced ROS generation.

### 3.4. The Protective Effects of High Glucose Based on Suppression of β1-Adrenoceptor Signaling

**Figure 5:**
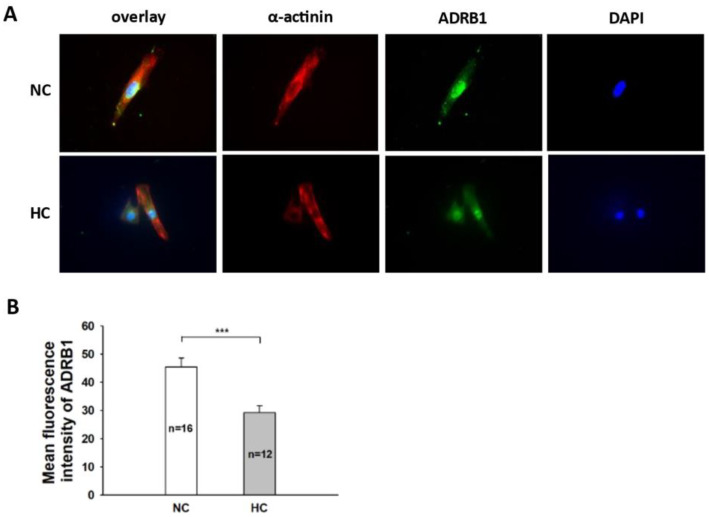
Immunofluorescence analysis of β1 (ADRB1) in hiPSC-CMs cultured with high concentration of glucose. “NC” represents data from hiPSC-CMs treated with normal level of glucose. “HC” represents data from hiPSC-CMs treated with a high level of glucose. (**A**) Immunofluorescence detecting the expressions of α-actinin (red) and ADRB1 (green). The cell nuclei were stained by DAPI (blue). (**B**) Statistical analyses of the fluorescence intensity representing the expression level of ADRB1. Data are presented as mean ± SEM and analyzed by *t*-test. The cell number of every experimental group is given as “n”. *** *p* < 0.001.

In order to examine whether the protective effects of glucose against Iso are mediated by changes in adrenoceptor proteins on the membrane of hiPSC-CMs, the immunofluorescence technique was applied to assess the expression of β1 (ADRB1). The expression level of ADRB1 on cell membrane was decreased significantly after the treatment with a high level of glucose (Figure 5).

**Figure 6:**
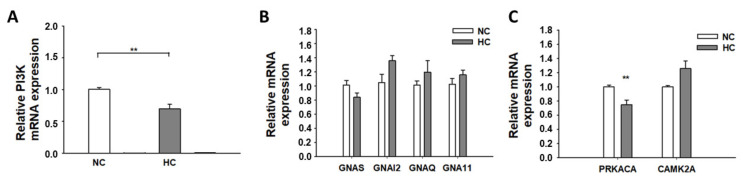
qPCR assay of PI3K, G protein, PKA, and CaMKII expression in hiPSC-CMs cultured with a high level of glucose. (**A**) The expression level of mRNA for PI3-kinase (PI3K) in hiPSC-CMs with normal (NC) and high glucose (HC). (**B**)The expression level of Gs (GNAS), Gi (GNAI2), Gq (GNAQ and GNA11) in hiPSC-CMs with normal (NC) and high glucose (HC). (**C**) The expression level of PKA (PRKACA) and CaMKII (CAMK2A) in hiPSC-CMs treated with normal (NC) and high concentration (HC) of glucose. Data are presented as mean ± SEM and analyzed by *t*-test. (n = 3, biological replicates). ** *p* < 0.01.

For the sake of further investigation of the potential mechanism in the downregulation of β-adrenoceptors on hiPSC-CMs membrane induced by a high level of glucose, we assessed insulin-PI3K signaling, which is an important pathway for the gene expression of many proteins, including adrenoceptors. The qPCR analysis showed that a high level of glucose could reduce the PI3K expression (Figure 6A), which has a possible correlation with the expression of β-adrenoceptors. This result may suggest the involvement of PI3K in the reduction in β-adrenoceptors induced by high glucose. It is also known that adrenoceptors are coupled to G-proteins (guanine nucleotide-binding proteins). We analyzed the mRNA levels of G-proteins including Gq, Gi, and Gs proteins and observed that the mRNA expression level of Gs was slightly decreased, whereas Gq and Gi were slightly increased after the treatment with a high level of glucose (Figure 6B). As β-adrenergic receptor stimulation and its downstream signaling via protein kinase A (PKA) and Ca^2+^/calmodulin-dependent protein kinase II (CaMKII) pathways are known to affect the cellular mechanisms of different cardiac pathological processes [21], we also analyzed the mRNA levels of PKA (PRKACA) and CaMKII (CAMK2A). It was detected that a high level of glucose could significantly reduce the PRKACA expression, but increased the CAMK2A expression (Figure 6C).

**Figure 7:**
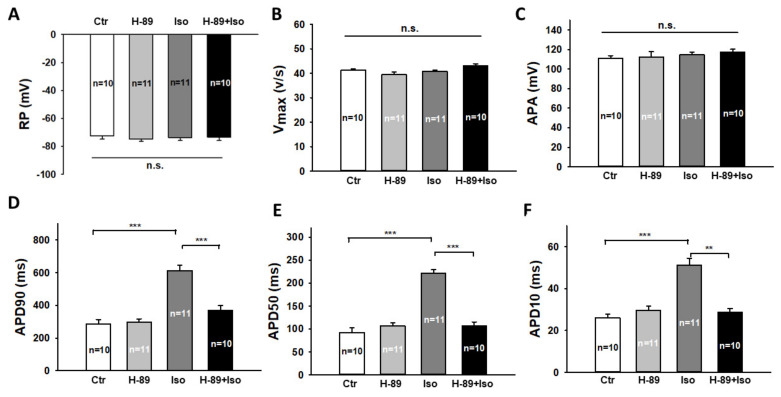
A PKA blocker attenuated the toxic effects of isoprenaline on action potentials in hiPSC-CMs. “Ctr” represents data from hiPSC-CMs treated with vehicle. “H-89” represents data from hiPSC-CMs treated with H-89 (10 µM). “Iso” represents data from hiPSC-CMs treated with a high concentration of Iso (1 mM). “H-89+Iso” represents data from hiPSC-CMs treated with H-89 plus a high concentration of Iso. Action potentials (AP) were recorded at 1 Hz stimulation. (**A**) Mean values of resting potentials (RP). (**B**) Mean values of maximal depolarization velocity of AP (Vmax). (**C**) Mean values of action potential amplitude (APA). (**D**) Mean values of action potential duration at 90% repolarization (APD90). (**E**) Mean values of action potential duration at 50% repolarization (APD50). (**F**) Mean values of action potential duration at 10% repolarization (APD10). Data are presented as mean ± SEM and analyzed by one-way ANOVA with Holm–Sidak post-test. The cell number of every experimental group is given as “n”. ** *p* < 0.01, *** *p* < 0.001. “n.s.” means no significance.

**Figure 8:**
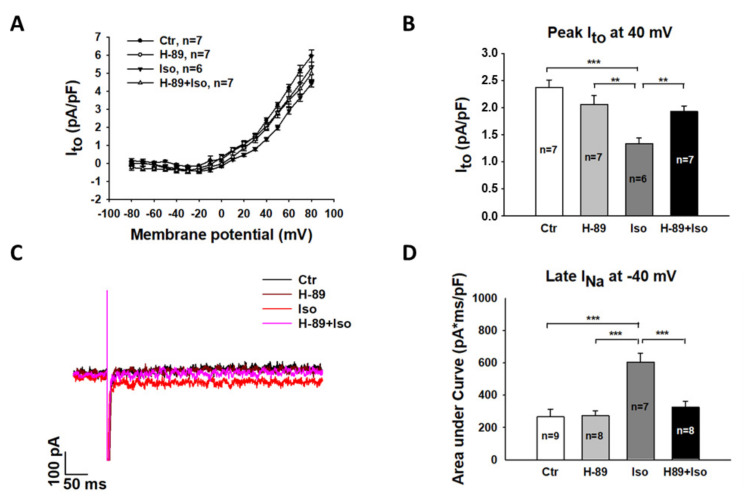
A PKA blocker attenuated the toxic effects of isoprenaline on transient outward currents (I_to_) and late sodium currents in hiPSC-CMs. “Ctr” represents data from hiPSC-CMs treated with vehicle. “H-89” represents data from hiPSC-CMs treated with H-89. “Iso” represents data from hiPSC-CMs treated with a high concentration of Iso. “H-89+Iso” represents data from hiPSC-CMs treated with H-89 and a high concentration of Iso. (**A**) I–V curves of I_to_ in cells of each group. (**B**) Mean values of peak I_to_ at 40 mV in cells of each group. (**C**) Representative traces showing late I_Na_ at −40 mV in cells of each group. (**D**) Mean values of late I_Na_ at −40 mV in cells of each group. The late I_Na_ was measured as the area under the current curve to zero line from 300 ms to 350 ms. Data are presented as mean ± SEM and analyzed by one-way ANOVA with Holm–Sidak post-test. The cell number of every experimental group is given as “n”. ** *p* < 0.01, *** *p* < 0.001.

The reduction in PKA expression induced by a high level of glucose suggested that PKA-blocker could mimic the effect of a high level of glucose against Iso. Thus, we pre-treated the cells with 10 µM H-89 dihydrochloride hydrate, a PKA-blocker, together with 1 mM Iso for 1 h and then checked action potential, late I_Na_, and I_to_. We found that H-89 abolished the prolongation of APD (Figure 7) and reversed the reduction in I_to_ (Figure 8A,B) and the increment in late I_Na_ (Figure 8C,D) induced by Iso. H-89 alone had no significant effect on late I_Na_ and I_to_. These data indicate that PKA is involved in the effects of high-concentration Iso.

### 3.5. The hiPSC-CMs from a Second (D2) and Third (D3) Donor Recapitulated Some Key Results in Cells from the First Donor (D1)

Given that all the results shown above were obtained from experiments in hiPSC-CMs from only one healthy donor (D1), some key experiments were repeated in hiPSC-CMs from the other two healthy subjects—donor 2 (D2) and donor 3 (D3). The effects of high-concentration Iso and a high level of glucose on AP parameters in hiPSC-CMs from D2 and D3 were very similar to those in hiPSC-CMs from D1 (Figures S6 and S7). These data imply that the individual variation is not large.

## 4. Discussion

### 4.1. Main Findings and Importance

In the present study, we observed that either a high concentration of Iso or a high level of glucose prolonged APD. Strikingly, we noticed that when both high-level Iso and high-level glucose were applied to cells, the APD-prolonging effect disappeared, suggesting that Iso and glucose counteracted each other.

Until the last decade, TTS was described as a benign reversible disease coupled with a favorable prognosis. However, according to recent updates in the published literature, TTS may have a poorer outcome than that assumed before [22]. It has been suggested that one of the main risk factors causing mortality in TTS patients is adverse rhythm disorders. The incidence of arrhythmias such as ventricular fibrillation has been reported to be around 7–14% [5,23,24]. Acquired LQTs, which is a major denominator of ventricular arrhythmias and sudden cardiac arrest in the general population, has been described as a predominant finding in these patients [23]. Therefore, it is of clinical importance to explore the pathogenic mechanisms of QT prolongation in TTS.

DM is an independent risk factor for sudden cardiac death and major complications of ACS [25]. QT prolongation is a well-established risk factor for life-threatening cardiac arrhythmias [26]. Patients with DM have a greater prevalence of QT prolongation than control populations [27,28], and acquired LQTs is an independent risk factor for cardiovascular death in diabetic people [29,30,31]. As QT prolongation may play a role in ventricular arrhythmias in both TTS and DM patients, it could be interesting and clinically relevant to assess whether and how the two diseases influence each other with respect to pathogenesis and clinical features. In the current study, we aimed to test our hypothesis that DM may protect cardiomyocytes from ion channel dysfunctions. For this, we employed hiPSC-CMs as the study platform because of the limited availability of human adult cardiomyocytes. We observed a counteraction of a high concentration of Iso and high-level glucose on action potentials and ion channel current (I_to_). The counteraction of Iso and glucose is on one side in agreement with the protective effect of DM against TTS, and on the other side is indicative of the protective effect of catecholamine against effects of high-level glucose. In this study, we focused on the protective effects of DM against TTS and the underlying mechanisms.

Regarding the mechanisms underlying the protective effects of DM against TTS, in addition to injury of nerves, another important point to be considered is an impairment of the adrenoceptor signaling in cardiomyocytes. Diabetic patients exhibit decreased exercise capacity, decreased sympathetic nerve activity, and decreased positive inotropic responses to β-adrenergic stimulation [32,33]. It is indicated that DM can impair the β-adrenoceptor signaling in cardiomyocytes in DM patients. Considering that β2-adrenergic receptors are only present in approximately 5% of ventricular cardiomyocytes [34], our study only investigated changes in β1-adrenergic receptor signaling. In our present study, the expression level of ADRB1 proteins on cell membranes detected by immunostaining was decreased significantly by high-level glucose. The immunofluorescence results showed that the protein levels of β1-receptors are in accordance with previous studies, but how β1-adrenoceptor expression was suppressed by high-level glucose needs to be addressed. Short-term stimulation of the β-adrenergic pathway in cultured cardiomyocytes can increase insulin-dependent glucose uptake through PKA-mediated Akt phosphorylation [35]. In contrast, long-term β-adrenergic stimulation can inhibit the absorption of insulin-dependent glucose in cultured cardiomyocytes [35]. On the other hand, hyperinsulinemia, which often occurs in patients with type 2 diabetes (T2D), may play a role in the dysregulation of β-adrenergic signal transduction in T2D, although the mechanism is not clear. Thus, it is suggested that there is a complex relationship between β-adrenergic and insulin signaling pathways in diabetic hearts, each of which plays an indispensable role in the development of cardiac pathology [36,37]. The downregulation of adrenoceptors in DM was explained by a compensatory action resulting from the hyperstimulation by norepinephrine in DM and HF [38,39,40]. Some studies have shown a decrease in insulin-induced PI3K/Akt, which can regulate β-adrenoceptor genes in heart preparations from diabetic mice [41,42]. Researchers also reported reduced cardiac PI3K/Akt insulin activation in T2D mice [43,44] and a porcine model of diet-induced obesity and insulin resistance [45]. These data are indicative of important roles of PI3K/Akt for β-adrenoceptor regulation. In addition, STAT1 and AP-1, two downstream factors of PI3K with a possible binding site of the β-adrenoceptor gene, were shown to be involved in estradiol-induced reduction in adrenoceptors [16]. With regard to the mechanisms underlying the reduction in adrenoceptor expression induced by high-level glucose treatment, in present study we observed that the expression of PI3K was reduced by high-level glucose without adrenergic (Iso) stimulation. This result may help to explain the changes in the downregulation of β adrenoceptors in DM.

G proteins are involved in regulating a variety of hormones and neurotransmitters. Activation of G protein-coupled receptors initiates cascades of signaling pathways, which modulate critical cardiac functions such as heart rate and contractility [46]. Iso stimulates ß1 receptor and activates Gs and then increases cAMP generation. The cAMP has at least two pathways—i.e., PKA and EPAC. EPAC has many downstream factors, including CAMKII. Both PKA and CAMKII can directly or indirectly regulate ion channel activity. In our present study, we checked different adrenergic receptor-related G proteins—namely, Gq, Gi, and Gs proteins in hiPSC-CMs treated with high-level glucose. Gs, which is coupled to β1-adrenoceptor, was slightly downregulated, whereas Gi and Gq were slightly upregulated, although the changes did not reach statistical significance. The downregulation of Gs may have contributed to the protective effects of high-level glucose against high-concentration Iso. Stimulation of β1-adrenergic receptor activates Gs protein and induces various types of signaling, including the PKA pathway in cardiac myocytes [47]. PKA plays an important role in increasing cardiac contractile activity and downstream signaling of β-adrenergic receptors through phosphorylation of a variety of proteins that regulate performance of the heart [48]. Our study showed that a high level of glucose could significantly reduce PKA but not CAMKII expression, which may explain why a high level of glucose attenuated the effects of Iso on APs and ion channel currents since PKA is a well-known regulator of different ion channels. Iso enhanced late I_Na_ and reduced I_to_ by stimulating ß1–Gs–PKA signaling, while high glucose suppressed this signaling and hence attenuated Iso effects. Whether the effects of Iso on late I_Na_ and I_to_ were through direct phosphorylation of sodium channel and KCND3 channel by PKA was not investigated in the current study and remains to be explored in future.

It is well known that high-concentration Iso and a high level of glucose can induce generation of ROS in cardiomyocytes. A previous study showed that enhanced ROS production induced by Iso may have contributed to ion channel dysfunction in the setting of TTS [16]. Therefore, we checked whether high-concentration Iso and high-level glucose influence each other in ROS production in hiPSC-CMs. We found that high-concentration Iso and high-level glucose both led to an elevation of ROS generation in cardiomyocytes, which is in line with previous reports. However, no counteraction between the Iso and glucose in ROS generation was observed. These results suggest that the high-level glucose-induced attenuation of effects of high-concentration Iso might not result from the change in ROS generation.

QT prolongation is due to the lengthening of the APD in cardiomyocytes. The APD is regulated by inward and outward ion currents. An increase in inward currents such as late I_Na_, I_Ca-L_, I_NCX_ or a decrease in outward currents such as I_to_, I_Kr_, I_Ks_ can prolong APD. The present study showed that high-concentration Iso prolonged APD and caused the changes in ion channel currents that are in agreement with the APD-prolongation. For example, the late I_Na_ was increased by Iso significantly and the outward K^+^ current’s I_to_ was reduced by Iso—both effects can prolong APD. The peak sodium channel current (I_Na_), which is the main determinant for Vmax of APs, was not influenced by Iso treatment, which can explain why Vmax was not changed by Iso in the study. To explore the reason for changes in ion channel currents, ion channel expression levels were investigated. The expression level of sodium channel contributing to late I_Na_ (SCN10A) was increased by high-concentration Iso, which is in agreement with late I_Na_ enhancement. The expression level of I_to_ channel (KCND3) was reduced by high-concentration Iso, which is also in agreement with I_to_ decrement. The result from immunofluorescence is inconsistent with the changes in I_to_ too. Taken together, high-concentration Iso enhanced SCN10A expression and suppressed KCND3 expression and in turn changed the inward and outward currents and prolonged APD. In the current study, high-level glucose also prolonged the APD, but with a smaller effect than that of high-concentration Iso. The high-level glucose reduced I_to_ and also KCND3 expression without significant effects on late I_Na_ or other currents measured in this study. Reduction in I_to_ may be partially responsible for the APD-prolongation caused by high-level glucose. Contributions of other ion channels that can influence APD to the APD-prolongation induced by high-level glucose cannot be excluded and need to be clarified in future studies.

An important and novel finding of this study is the counteraction of high-level glucose and high-concentration Iso on APD. On the ion channel current level, we also observed the counteraction on I_to_. This may explain partially the counteraction on APD-prolongation. On late I_Na_, a protective effect of high-level glucose against high-concentration Iso was detected. This also supports our hypothesis that high-level glucose may attenuate toxic effects of Iso on ion channel functions. To connect the changes in APD and ion channel currents with changes in β1-adrenoceptor signaling in presence of high-concentration Iso, we finally checked the ion channel current changes in cardiomyocytes treated with H-89, which is a PKA-blocker [49]. We found that H-89 led to elimination of the Iso-induced APD-prolongation, enhancement of late I_Na_, and the decrement of I_to_, implying that PKA mediated Iso-effects via phosphorylation of the channels or other proteins that can regulate the channels.

Taking the data together, high-level glucose reduced β1-adrenoceptor, Gs-protein, and PKA expression and hence suppressed the effects of Iso on channel currents (late I_Na_ and I_to_) and APD. In addition, PI3K/Akt reduction caused by high-level glucose may contribute to the downregulation of the β1-adrenoceptor. Although data regarding signaling of the ß1-receptor are preliminary and more studies are required for clarifying the concrete mechanisms underlying the effects of toxic Iso and high glucose, this study can provide some useful information. The study revealed that the QTc prolongation in both TTS and DM patients may result from the APD prolongation-caused alterations of late I_Na_ and I_to,_ which provides information for understanding the clinical phenomenon and potential therapeutic targets for treating or preventing arrhythmias resulting from QTc prolongation in TTS or DM patients; high glucose can attenuate toxic effects of catecholamine via reducing ß1-receptor level or the receptor-related signaling, which adds novel information for understanding why in DM patients the risk of TTS is reduced.

### 4.2. Conclusions

This study demonstrated that a high level of glucose may exert protective effects against catecholamine excess through β1-adrenoceptor signaling. High-concentration Iso and a high level of glucose caused ion channel dysfunctions including increased late I_Na_ and decreased I_to_ and prolonged APD, which reflects the long QT phenomenon in DM or TTS patients. A high level of glucose might reduce the sensitivity of cardiomyocytes to catecholamine through a reduction in adrenoceptor expression and inhibition of the PKA pathway and thereby might reduce the toxic effects of catecholamine excess and the occurrence of arrhythmias in TTS.

### 4.3. Study Limitations

In regard to this study, some limitations should be considered. Firstly, β-adrenoceptor contains several subtypes: β1, β2, and β3. This study did not investigate whether the roles of the β2 and β3 subtypes might be responsible for the changes. Secondly, although hiPSC-CMs possess similarities to adult human cardiomyocytes, they demonstrate differences when compared together. We cannot exclude the possibility that some physiological properties in hiPSC-CMs might be different from those in adult human cardiomyocytes. Moreover, human ventricular cardiomyocytes were not used as a comparison in this study due to the limitation of access to the cells. Thirdly, the study did not use hiPSC-CMs from TTS patients. Thus, whether hiPSC-CMs from TTS patients display different characteristics still needs to be explored. Fourthly, because of the lower sensitivity of hiPSC-CMs to catecholamine compared with adult human cardiomyocytes, the concentration of Iso (1 mM) used in vitro is much higher than the catecholamine concentration in TTS patients. Therefore, it cannot be ruled out that native cardiomyocytes in vivo respond differently to catecholamine from those in the study. Further in vivo studies may be needed to prove the findings from this study. Moreover, high-concentration Iso and high-level glucose together showed counteractive effects on APD and channel current (Ito). We investigated only the protective effects of high-level glucose against high-concentration Iso. How high-concentration Iso counteracts the high-level glucose effects on ion channels and APD was not assessed and needs to be clarified by further studies.

## Data Availability

All the data reported in this study are contained in the paper and supplemental data.

## Supplementary Materials

The following supporting information can be downloaded at: https://www.mdpi.com/article/10.3390/jcdd9030076/s1, Figure S1: title Figure S1. Effects of high concentration isoprenaline and high level of glucose on L-type calcium channel currents; Figure S2. Effects of high concentration isoprenaline and high level of glucose on Na/Ca exchanger current (I_NCX_); Figure S3. Effects of high concentration isoprenaline and high level of glucose on rapidly activating delayed rectifier current (I_Kr_) and slowly activating delayed rectifier current (I_Ks_); Figure S4. High concentration isoprenaline and high level of glucose changed ion channel expression levels; Figure S5. Immunofluorescence analysis of KCND3 protein in hiPSC-CMs; Figure S6. Effects of high concentration isoprenaline and high level of glucose on APs in hiPSC-CMs from donor 2; Figure S7. Effects of high concentration isoprenaline and high level of glucose on APs in hiPSC-CMs from donor 3.
