# Supplementary material for: Glucose Counteracts Isoprenaline Effects on Ion Channel Functions in Human-Induced Pluripotent Stem Cell-Derived Cardiomyocytes
Source: J Cardiovasc Dev Dis. 2022 Mar 4;9(3):76. doi: 10.3390/jcdd9030076 (PMC8955312; doi:10.3390/jcdd9030076)
Supplement: Supplementary file 1 [file jcdd-09-00076-s001.zip › jcdd-1589557-supplementary.pdf]

Supplementary material

Glucose counteracts isoprenaline effects on ion channel functions in human-induced pluripotent stem cell-derived cardiomyocytes

Lin Qiao[1], Xuehui Fan[1,2,3], Zhen Yang[1], Ibrahim El-Battrawy[1,3], Xiaobo Zhou[1,2,3] and Ibrahim Akin[1,3]

[1]First Department of Medicine (Department of Cardiology, Angiology, Haemostaseology and Medical Intensive Care), Medical Faculty Mannheim, University Medical Centre Mannheim (UMM), University of Heidelberg, Mannheim, Germany

[2]Key Laboratory of Medical Electrophysiology, Ministry of Education and Medical Electrophysiological Key Laboratory of Sichuan Province, Collaborative Innovation Center for Prevention of Cardiovascular Diseases, Institute of Cardiovascular Research, Southwest Medical University, Luzhou, Sichuan, China

[3]European Center for AngioScience (ECAS) and German Center for Cardiovascular Research (DZHK) partner site Heidelberg/Mannheim, Mannheim, Germany

Address for correspondence:

Xiaobo Zhou, MD.

First Department of Medicine, University Medical Centre Mannheim,

Theodor-Kutzer-Ufer 1-3, 68167 Mannheim, Germany.

Phone: 0049-621-383-1448. Fax: 0049-621-383-1474.

E-mail: xiaobo.zhou@medma.uni-heidelberg.de.

Figure legends

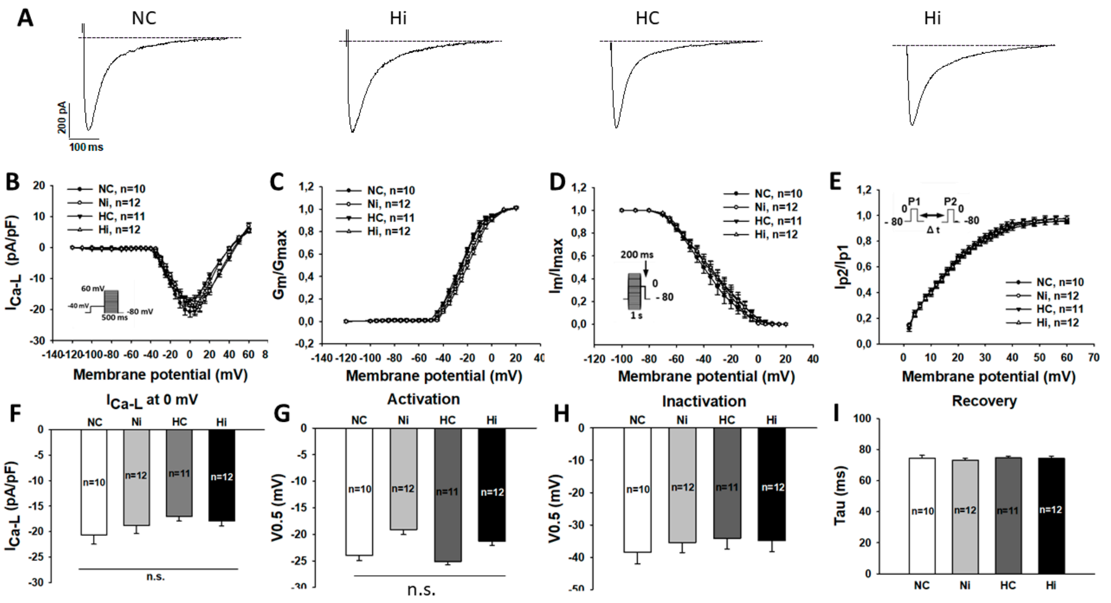

Figure S1. Effects of high concentration isoprenaline and high level of glucose on L-type calcium channel currents. "NC" represented data from hiPSC-CMs treated with normal level of glucose. "Ni" represented data from hiPSC-CMs treated with normal level of glucose and high concentration of Iso. "HC" represented data from hiPSC-CMs treated with high level of glucose. "Hi" represented data from hiPSC-CMs treated with high level of glucose and high concentration of Iso. L-type calcium channel currents ($I_{Ca\text{-}L}$) were recorded using protocols shown in B, D and E (inset). The (A) Representative traces of peak $I_{Ca\text{-}L}$ at 0 mV in cells from each group. (B) I-V curves of peak $I_{Ca\text{-}L}$ in cells of each group. (C) Activation curves of peak $I_{Ca\text{-}L}$ in cells of each group. (D) Inactivation curves of peak $I_{Ca\text{-}L}$ in cells of each group. (E) Peak $I_{Ca\text{-}L}$ curves

of recovery from inactivation in cells of each group. (F) Mean values of peak I_{Ca-L} at 0 mV. (G) Mean values of voltage at 50% activation. (H) Mean values of voltage at 50% inactivation. (I) Mean values of time constant (Tau) of recovery from inactivation. Data were presented as mean ± SEM and analyzed by one-way ANOVA with Holm-Sidak post-test. The cell number of every experiment group was given as "n". "n.s." means no significance.

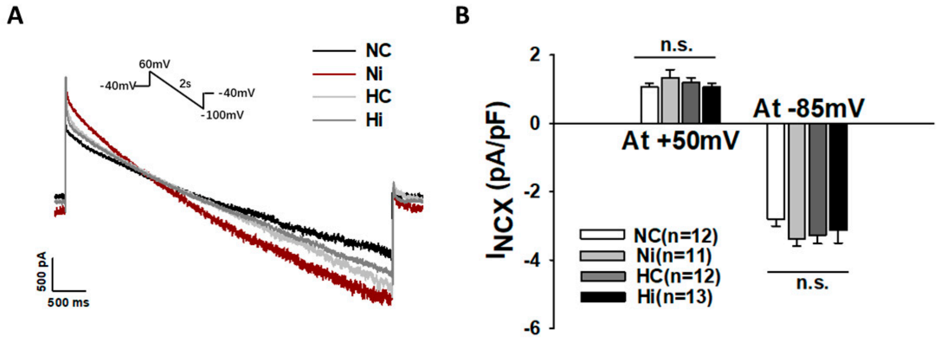

Figure S2. Effects of high concentration isoprenaline and high level of glucose on Na/Ca exchanger current (I_{NCX}). "NC" represented data from hiPSC-CMs treated with normal level of glucose. "Ni" represented data from hiPSC-CMs treated with normal level of glucose and high concentration of Iso. "HC" represented data from hiPSC-CMs treated with high level of glucose. "Hi" represented data from hiPSC-CMs treated with high level of glucose and high concentration of Iso. The Ca/Na exchanger current (I_{NCX}) was recorded using protocols shown in A (inset). (A) Representative traces of I_{NCX} in cells

from each group. (B) Mean values of I_{NCX} at 50 mV and -85 mV. Data were presented as mean ± SEM and analyzed by one-way ANOVA with Holm-Sidak post-test. The cell number of every experiment group was given as "n". "n.s." means no significance.

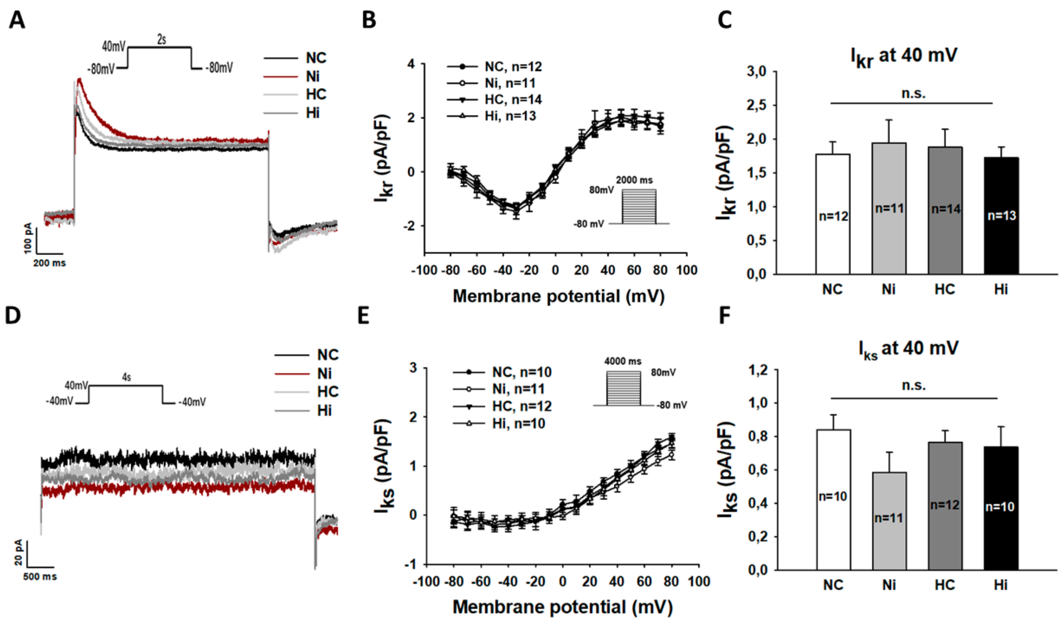

Figure S3. Effects of high concentration isoprenaline and high level of glucose on rapidly activating delayed rectifier current (I_{Kr}) and slowly activating delayed rectifier current (I_{Ks}). "NC" represented data from hiPSC-CMs treated with normal level of glucose. "Ni" represented data from hiPSC-CMs treated with normal level of glucose and high concentration of Iso. "HC" represented data from hiPSC-CMs treated with high level of glucose. "Hi" represented data from hiPSC-CMs treated with high level of glucose and high concentration of Iso. The rapidly activating delayed rectifier current (I_{Kr}) or slowly activating delayed rectifier current (I_{Ks}) was recorded using

protocols shown in A-B and D-E (inset). (A) Representative traces of I_{Kr} in cells from each group. (B) I-V curves of of I_{Kr} in cells from each group. (C) Mean values of peak I_{Kr} at 40 mV in cells from each group. (D) Representative traces of I_{Ks} in cells from each group. (E) I-V curves of of I_{Ks} in cells from each group. (F) Mean values of peak I_{Ks} at 40 mV in cells from each group. Data were presented as mean ± SEM and analyzed by one-way ANOVA with Holm-Sidak post-test. The cell number of every experiment group was given as "n". "n.s." means no significance.

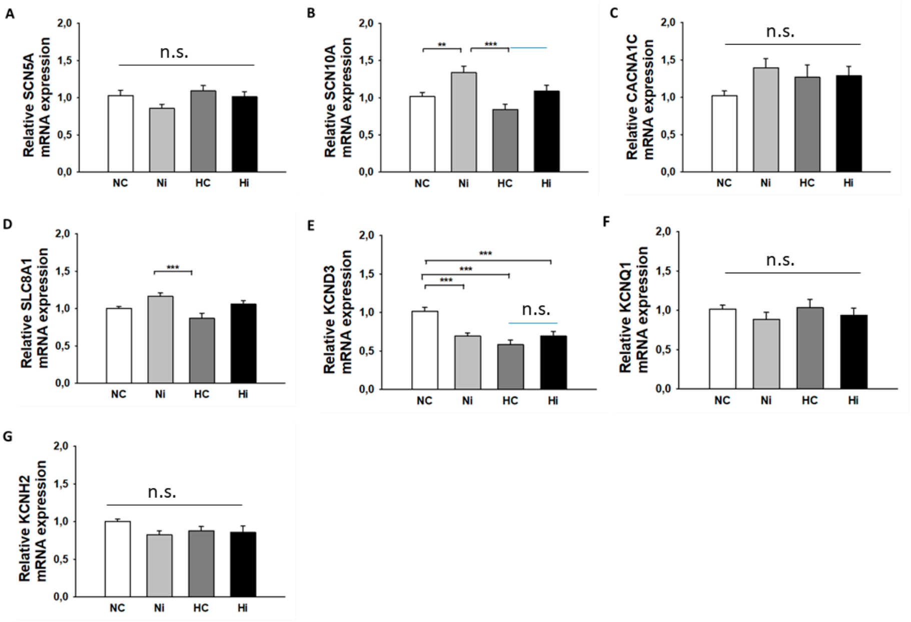

Figure S4. High concentration isoprenaline and high level of glucose changed ion channel expression levels. "NC" represented data from hiPSC-CMs treated with normal level of glucose. "Ni" represented data from hiPSC-CMs treated with normal level of glucose and high concentration of Iso. "HC" represented data from hiPSC-CMs treated with high level of glucose.

"Hi" represented data from hiPSC-CMs treated with high level of glucose and high concentration of Iso. qPCR analysis was performed to measure the expression levels of ion channels. (A) Mean values of cardiac sodium channel Nav1.5 (SCN5A) expression. (B) Mean values of sodium channel Nav1.8 (SCN10A) expression. (C) Mean values of L-type calcium channel (CACNA1C) expression. (D) Mean values of Na^+/Ca^{2+} exchanger (SLC8A1) expression. (E) Mean values of transient outward K^+ channel (KCND3, I_{to}) expression. (F) Mean values of slowly activating delayed rectifier K^+ (KCNQ1, I_{Ks}) channel expression. (G) Mean values of channel rapidly activating delayed rectifier K^+ (KCNH2, I_{Kr}) expression. Data were presented as mean ± SEM and analyzed by one-way ANOVA with Holm-Sidak post-test. (n=4, biological replicates). **P<0.01, ***P<0.001. "n.s." means no significance.

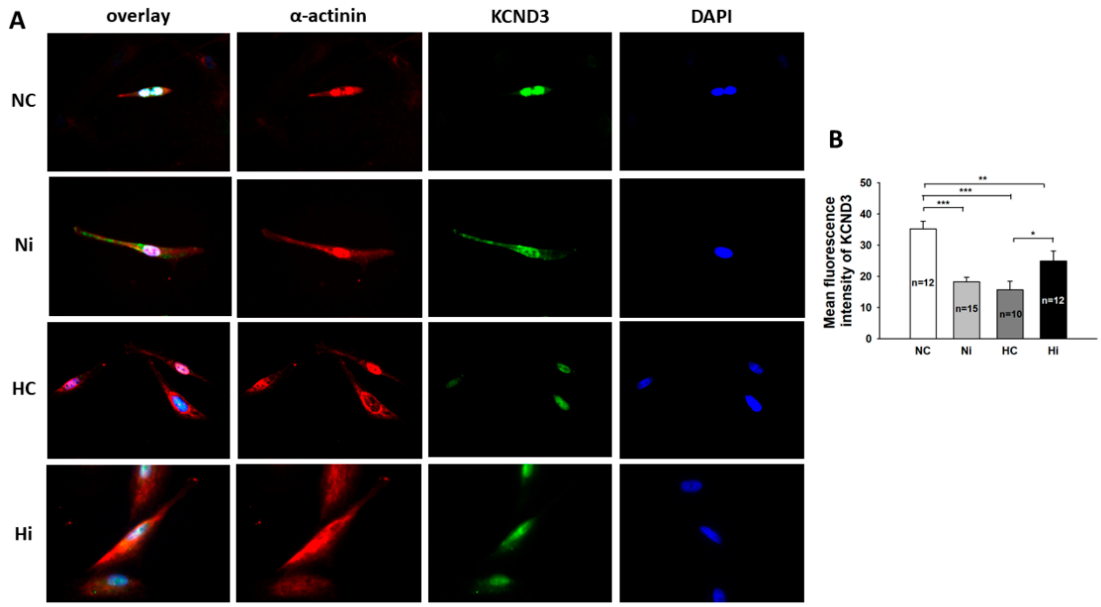

Figure 5. Immunofluorescence analysis of KCND3 protein in hiPSC-CMs.

"NC" represented data from hiPSC-CMs treated with normal level of glucose. "Ni" represented data from hiPSC-CMs treated with normal level of glucose and high concentration of Iso. "HC" represented data from hiPSC-CMs treated with high level of glucose. "Hi" represented data from hiPSC-CMs treated with high level of glucose and high concentration of Iso. (A) Immunofluorescence detecting the expressions of α-actinin (red) and KCND3 (green). The cell nuclei were stained by DAPI (blue). (B) Statistical analyses of the fluorescence intensity representing the expression level of KCND3 protein. Data were presented as mean ± SEM and analyzed by one-way ANOVA with Holm-Sidak post-test. The cell number of every experiment group was given as "n". *P<0.05, **P<0.01, ***P<0.001.

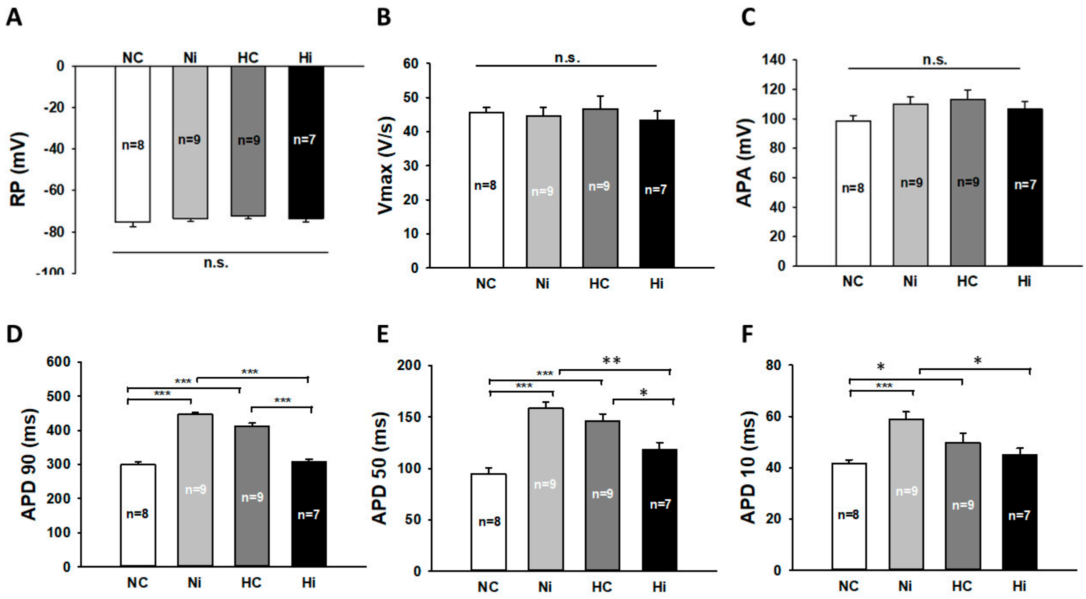

Figure S6. Effects of high concentration isoprenaline and high level of glucose on APs in hiPSC-CMs from donor 2. APs were recorded in

hiPSC-CMs from donor 2 (D2) under the same condition as that in donor 1 (D1) cells. "NC" represented data from hiPSC-CMs treated with normal level of glucose. "Ni" represented data from hiPSC-CMs treated with normal level of glucose and high concentration of Iso. "HC" represented data from hiPSC-CMs treated with high level of glucose. "Hi" represented data from hiPSC-CMs treated with high level of glucose and high concentration of Iso. Action potentials (AP) were recorded at 0,5 Hz stimulation. (A) Mean values of resting potentials (RP). (B) Mean values of maximal depolarization velocity of AP (Vmax). (C) Mean values of action potential amplitude (APA). (D) Mean values of action potential duration at 90% repolarization (APD90). (E) Mean values of action potential duration at 50% repolarization (APD50). (F) Mean values of action potential duration at 10% repolarization (APD10). Data were presented as mean ± SEM and analyzed by one-way ANOVA with Holm-Sidak post-test. The cell number of every experiment group was given as "n". *P<0.05, **P<0.01. ***P<0.001. "n.s." means no significance.

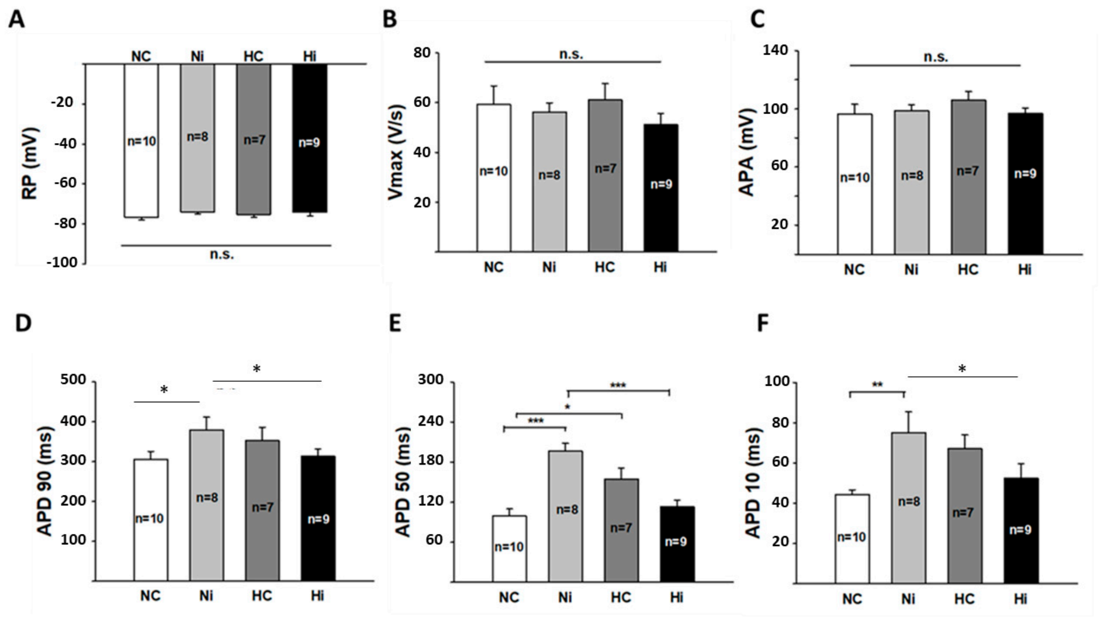

Figure S7. Effects of high concentration isoprenaline and high level of glucose on APs in hiPSC-CMs from donor 3. APs were recorded in hiPSC-CMs from donor 3 (D3) under the same condition as that in donor 1 (D1) cells. "NC" represented data from hiPSC-CMs treated with normal level of glucose. "Ni" represented data from hiPSC-CMs treated with normal level of glucose and high concentration of Iso. "HC" represented data from hiPSC-CMs treated with high level of glucose. "Hi" represented data from hiPSC-CMs treated with high level of glucose and high concentration of Iso. Action potentials (AP) were recorded at 0,5 Hz stimulation. (A) Mean values of resting potentials (RP). (B) Mean values of maximal depolarization velocity of AP (Vmax). (C) Mean values of action potential amplitude (APA). (D) Mean values of action potential duration at 90% repolarization (APD90). (E) Mean values of action potential duration at 50% repolarization (APD50). (F) Mean values of action potential duration at 10% repolarization (APD10). Data were presented as mean ± SEM and analyzed by one-way ANOVA with

Holm-Sidak post-test. The cell number of every experiment group was given as “n”. *P<0.05, **P<0.01. ***P<0.001. “n.s.” means no significance.
